# Predicting olfactory receptor neuron responses from odorant structure

**DOI:** 10.1186/1752-153X-1-11

**Published:** 2007-05-04

**Authors:** Michael Schmuker, Marien de Bruyne, Melanie Hähnel, Gisbert Schneider

**Affiliations:** 1 Johann Wolfgang Goethe Universität, Beilstein Endowed Chair for Cheminformatics, Institute of Organic Chemistry and Chemical Biology, Siesmayerstr. 70, 60323 Frankfurt am Main, Germany; 2 Freie Universität Berlin, Institute of Biology-Neurobiology, Königin-Luise-Str. 28–30, 14195 Berlin, Germany; 3 Monash University, School of Biological Sciences, Wellington Road, Clayton VIC 3800, Australia; 4 (present address) Freie Universität Berlin, Institute of Biology-Neurobiology, Königin-Luise-Str. 28–30, 14195 Berlin, Germany

## Abstract

**Background:**

Olfactory receptors work at the interface between the chemical world of volatile molecules and the perception of scent in the brain. Their main purpose is to translate chemical space into information that can be processed by neural circuits. Assuming that these receptors have evolved to cope with this task, the analysis of their coding strategy promises to yield valuable insight in how to encode chemical information in an efficient way.

**Results:**

We mimicked olfactory coding by modeling responses of primary olfactory neurons to small molecules using a large set of physicochemical molecular descriptors and artificial neural networks. We then tested these models by recording *in vivo *receptor neuron responses to a new set of odorants and successfully predicted the responses of five out of seven receptor neurons. Correlation coefficients ranged from 0.66 to 0.85, demonstrating the applicability of our approach for the analysis of olfactory receptor activation data. The molecular descriptors that are best-suited for response prediction vary for different receptor neurons, implying that each receptor neuron detects a different aspect of chemical space. Finally, we demonstrate that receptor responses themselves can be used as descriptors in a predictive model of neuron activation.

**Conclusion:**

The chemical meaning of molecular descriptors helps understand structure-response relationships for olfactory receptors and their "receptive fields". Moreover, it is possible to predict receptor neuron activation from chemical structure using machine-learning techniques, although this is still complicated by a lack of training data.

## Introduction

Olfactory Receptors (ORs) encode chemical stimuli in neuronal activity. The gene family of ORs consists of G-protein coupled receptors (GPCRs) and was first described for rats [1]. In *Drosophila*, the organism we considered in this study, as well as in mammals and vertebrates in general, each Olfactory Receptor Neuron (ORN) carries one type of OR [2], such that the response of each ORN to a chemical substance is mainly determined by the receptor it expresses [3].

The fact that there is no crystal structure available for any OR hampers structure-based approaches such as automated molecular docking to examine ligand binding characteristics. Although attempts have been made to use models based on homology to rhodopsin [4-6], these approaches suffer from the cumbersome creation of such a model and the remaining errors inherent to homology modeling [7,8].

Araneda and cowokers pursued a ligand-based approach to characterize the rat's I7 OR [9]. By testing a large number of ligands, they were able to establish a verbal characterization of preferred I7 ligands in terms of functional groups and carbon chain length and rigidity. Such an approach however only provides qualitative data for a limited number of odorants. It does not describe ORN tuning in quantifiable parameters that can be determined for any chemical.

Here we present a method providing an objective way of predicting ORN responses to arbitrary odorants. We have developed a model that uses a distinct set of physicochemical parameters to describe the structure of odor molecules and predict their activity at *Drosophila *receptors.

We followed a classic approach to derive Structure-Activity-Relationships (SARs) by calculating molecular descriptors and training Artificial Neural Networks (ANNs), as it has been applied in other studies to characterize ligand affinity to specific receptors [10-12]. Similar techniques were previously used to model human psychophysical data, i.e. odor and aroma characteristics [13-16]. However, odor percepts are the result of a nonlinear transformation of ORN inputs in the brain and do not necessarily reflect OR properties [17]. By contrast, we restricted our study to modeling receptor responses, because these are more likely to be dominated by physicochemical properties of the odorants, assuming OR activation is the result of ligand-receptor binding through intermolecular interactions.

In addition, we suggest that quantifying the molecular properties relevant for activating olfactory receptors reveals how chemical space is encoded by the receptor repertoire of a specific organism. One may assume that such an array of ORs has evolved to provide a useful representation of chemical space through an efficient coding scheme. Determining the actual properties of the chemical world that are detected by ORs may thus provide an efficient way to represent molecules in a computational framework in general.

## Results and discussion

The goal of our study was twofold: First, we aimed at predicting ORN responses from molecular structure. Second, we wanted to describe structure-activity relationships between the odorant and the activated receptor.

To achieve the first aim, we trained artificial neural network models on an existing dataset of ORN responses, using selected subsets of chemical descriptors for odorant representation. We then recorded the responses of these same ORNs to a new set of chemicals to test whether the models we generated can be used to predict an odorant's activity.

With the second aim in mind we analyzed the set of discriminative descriptors in order to characterize chemical properties that favor activation of each ORN.

### Modeling ORN response and testing

We trained ANNs to model the activity of seven *Drosophila *ORNs in response to stimulation with odorant molecules. As training data we used the responses of *Drosophila *ORNs to 47 odorants that were measured by electrophysiological *in vivo *recordings in a previous study [18]. Structure drawings of these 47 odorants are depicted in the supplemental material [see Additional file 1], as well as their names and the activity values (in spikes/s) [see Additional file 2]. We defined thresholds in activity such that a given compound can be classified as either "active", "inactive", or "uncertain", depending on the spike rate it elicits in the ORN. Compounds with uncertain activity were not used for training the ANNs for that specific ORN. After selecting relevant descriptors for each ORN (*cf*. next section), we trained 30,000 ANN models per ORN, selected those with the highest predictive power, and used them to predict ORN responses to 21 compounds, which were subsequently tested *in vivo *(in the following referred to as "test data"). We also assayed ten compounds that had already been tested in the previous study [18]. The compounds in the test data set are depicted in the supplement [see Additional file 3].

For analysis of the results we transformed spike rates using the same thresholds as we used for the training data. As in the training data, molecules with spike rates between the upper and lower threshold were excluded from the analysis for the respective ORN.

We assessed prediction performance using the Matthews Correlation Coefficient for binary data (MCC, eq. 4). Table 1 shows the MCC for the training data and the tested data. We excluded ethyl-3-hydroxybutyrate at ab2B and butyl acetate at ab3A from the calculation of the MCC of the test set, since these molecules were used to select the best models (see Experimental section for details). These compounds have entered the modeling process prior to testing and hence are not valid "test" compounds for those ORNs.

**Table 1:** Matthew's Correlation Coefficient (MCC) for training and test. The upper row refers to the performance on the training data, the lower row to performance on the test data

ORN		ab1D	ab2A	ab2B	ab3A	ab3B	ab5B	ab6A
MCC	training	1.00	1.00	1.00	1.00	0.77	1.00	0.86
	test	0.69	0.69	0.17	0.85	0.34	0.68	0.66

Five out of seven models succeeded in correctly predicting the training data. The predictions for the ab3B and 6A neuron show imperfect performance, but still correlate with the activity in the training data. The prediction of ORN response to novel molecules shows a mixed picture: For the ab3A ORN, the model achieved an MCC of 0.85, providing reliable prediction. For the ab1D, 2A, 5B and 6A ORNs, the MCCs range from 0.66 to 0.69, still indicating good performance. In contrast, the models showed only weak performance predicting activity for the ab2B (MCC = 0.17) and 3B ORNs (MCC = 0.34).

The discrepancy between performance on the training data and the test data for some receptors may have several causes. First, although we used cross-validated training and in some cases additional activity data for model selection, due to the large number of models we built, it is possible that some models perfectly predict all training data, albeit by chance. Second, descriptor selection was performed on the whole data set instead of a cross-validated procedure, possibly "over-optimizing" descriptor space for the training data. However, because of the data splitting necessary for cross-validation, the number of data instances in one part of the data would have been too small for the statistical test we used to select descriptors. In both cases, the performance on the independent test set reveals the actual quality of prediction. This set contained only substances that did not enter the model creation at any point and is thus not affected by the above issues.

The supplement gives detailed insight into the compounds we used for testing and the results of the screening, in comparison with the predictions [see Additional file 4]. It should be noted that one compound (cyclohexanone) was inactive at ab3A in the training data (3 spikes/s), but active in the test data (33 spikes/s). A similar observation was made for 4-methylphenol at the ab1D neuron: its activity was uncertain in the training data (22 spikes/s), but it was inactive in test data (5 spikes/s). These differences may be a consequence of the effect that minimal variations in concentration may suffice to elicit a response [18].

A possible source of error in the predictions is that it is not always certain that the compound actually arriving at the receptor neuron did not undergo degradation, or that traces of other compounds contaminated the stimulus, for example as by-products from synthesis or as remnants after purification. These effects cannot be addressed by this study, but would require analysis of the air stream in parallel to the measurements, for example by gas chromatography [19,20].

One point of discussion is the threshold setting for activity assignment, in that it followed no algorithmic procedure. However, these thresholds proved to be sensible choices, and appeared reasonable to us according to the data. First of all, the application of thresholds was necessary to simplify the data. As in any modeling study, simplifications have to be introduced in order to focus on the most relevant features, especially when the amount of data is limited. In this case, we chose to discard the quantitative activity data in favor of a binary active/inactive prediction. Although our threshold settings may have enhanced the aforementioned difference in activity assignment, these were more likely due to changes in the experimental setup, or variance in the *Drosophila *stock between the measurements of the training and test sets. Further, the models do not take into account the different vapor pressures of the compounds or effects of dose dependency of the responses, because the required data was not available for all compounds. We also did not address any possible effects of modifiers of OR activity such as Olfactory Binding Proteins (OBPs). These proteins populate the aqueous lymph surrounding olfactory dendrites and have been shown to be involved in olfaction. *Drosophila *mutants devoid of the LUSH OBP have defects in avoiding high alcohol concentrations [21] and lack response to a pheromone [22]. It has also been suggested that OBPs are involved in shuttling hydrophobic odorants through the lymph [23]. The model, being trained on the activation data of ORNs in their "native surround" (i.e. the lymph), implicitely treats everything between the odorant and ORN activation as a "black box" and hence also contains effects of OBPs, if present.

### Interpretation of descriptor selection

As stated above, we selected subsets of descriptors that are best suited for separating active from inactive compounds prior to ANN training. In addition to reducing the "noise" introduced into the data by unsuitable descriptors, the ranked list of descriptors can also give insight into the SAR of the ORNs. Since each descriptor represents a molecular feature, descriptors in the selected subset point to potentially preferred molecular features detected by an ORN. The sum of preferred features determines an ORN's "receptive field".

The descriptor rankings were produces using the *p*-value from a Kolmogorov-Smirnov test (KS-test) for significant difference between two data sets (inactive vs. active compounds), separately for each ORN. Descriptors with the lowest *p*-values were ranked highest. The ranked lists of descriptors including their associated *p*-values are given in the supplement [see Additional file 5].

We observed that the set of highest ranking descriptors is different for each ORN. This may correspond to a different SAR for each ORN, in that different chemotypes are recognized by different receptors. In the following, we describe how the descriptor rankings relate to the SARs of the ORNs in this study. For the sake of brevity, we refer to individual descriptors by their abbreviations. More elaborate explanations of all descriptors that appear here and in the ranked lists are provided in the supplement [see Additional file 6].

#### ab1D

**Figure 1:**
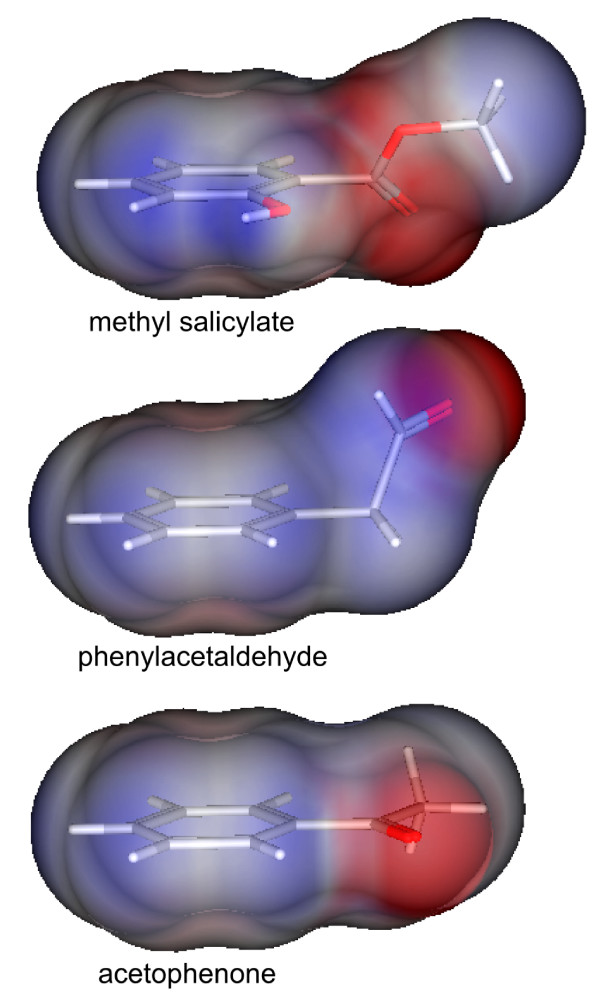
**Disk-like shapes of ab1D activators**. Three activators of ab1D, methyl salicylate (187 spikes/s), phenylacetaldehyde (76 spikes/s) and acetophenone (157 spikes/s) show their disk-like shape in surface representation. Red areas indicate negative partial charge, blue areas positive partial charge, and white indicates neutral (= no) charge.

For ab1D, the highest ranked descriptor is std_dim3, a 3D shape descriptor that describes the standard deviation along the principal component axis of the atom coordinates. Typical activators of ab1D (methyl salicylate, acetophenone, phenylacetaldehyde) have disk-like shape in common due to their aromatic ring systems (see Figure 1). Hence, they will have small values for this descriptor, discriminating them from the other molecules in the data set. This descriptor does not feature strongly in the rankings of other ORNs that respond to aliphatic compounds. Furthermore, the high ranking of several descriptors for charge distribution on the molecular surface (such as PEOE_VSA_FPNEG, Q_VSA_FNEG, FCASA-) reflect the exposed carbonyl groups in most activators of ab1D, creating a focused negative partial charge distribution on the molecular surface (cf. Figure 1). Charge distribution descriptors feature high on the list of several ORNs.

#### ab2A

**Figure 2:**
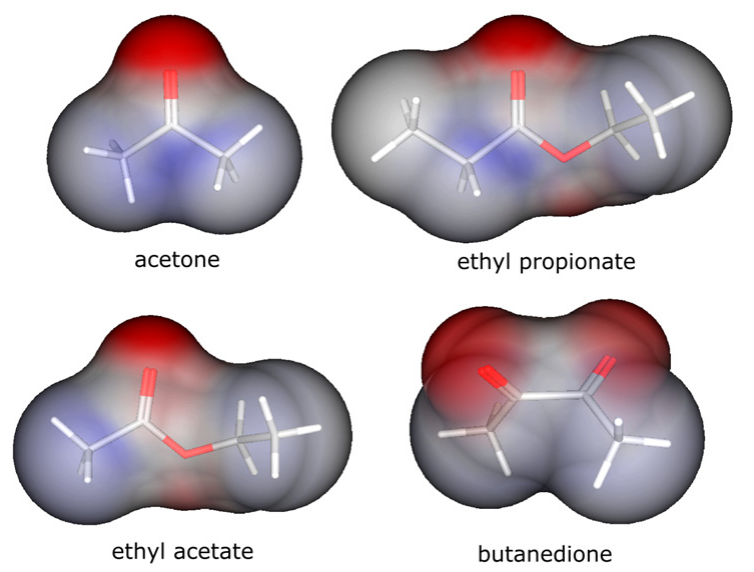
**Charge distribution of ab2A activators**. Conolly-surface representation for activators of the ab2A ORN. Color scheme is identical to Figure 1.

A strong effect of partial charge can also be observed for the activators of the ab2A ORN (ethyl acetate, 2,3-butanedione, propanone, ethyl propionate), which are all comparably small and bear a focused negative partial charge on the molecular surface (cf. Figure 2). The focused charge is again represented in the highest scoring PEOE_VSA_FPNEG descriptor. The high rank of a_ICM can be related to the small molecule size. It describes the mean atom information content, which reflects the *entropy*, used by its information-theoretical meaning, in atom composition. For two equal-sized molecules, the one which is composed of more different atom types will have the higher entropy. Accordingly, for two molecules with the same number of different atom types, the smaller one will have higher entropy. Now the high scoring molecules incorporate only two atom types, namely O and C, as well as the majority of the remaining molecules in the data set. Thus, the smaller molecule size likely is the discriminating feature. Several connectivity descriptors (chi1v_C, chi1_C etc.) also reflect the importance of molecule size.

#### ab2B and ab3A

The AM1_HOMO descriptor, which is an index for "reactivity", yields a high rank for the ab3A neuron. Moreover, the MNDO_HF descriptor (heat of formation) correlates well with ab3A spike rate change (Pearson correlation coefficient: -0.55, *p *< 10^-4^). Also, the ionization potential (reflected in the AM1_IP, PM3_IP and MNDO_IP descriptors) yields a high rank. All these descriptors relate to the reactivity of a molecule and are negatively correlated with activity. This seems evident if one considers that most activators of ab3A are esters, which are less reactive than for example aldehydes and primary alcohols, two groups to which many of the non-activators belong.

Similar observations can be made for ab2B, where four of the five activators of the ab2B ORN (ethyl butanoate, hexanol, *γ*-valerolactone, ethyl-2-methylbutanoate) have a slightly elevated ionization potential according to the AM1_IP descriptor, compared to non-activators (e.g. 3-methylthio-1-propanol, benzaldehyde or linalool), as well as a high ranking of the AM1_HOMO descriptor.

#### ab5B

For the ab5B ORN, the highest ranked descriptors are related to molecular shape, expressed by the descriptors developed by Kier & Hall descriptors [24] (KierA3, KierA1, KierA2, KierFlex, Kier2, Kier3). In combination with the high ranked b_1rotR descriptor (the relative number of rotatable bonds in the molecule), this reflects ab5B's preference for larger, flexible ligands, such as pentyl acetate, 2-heptanone and 3-octanol.

#### ab6A

Finally, for the ab6A ORN the *κ*3 and *κ*2 descriptors described by Kier & Hall [24] rank highest (Kier3 and Kier2). *κ*2 encodes information about the "spatial density of atoms" in a molecular graph, while *κ*3 encodes the "centrality of branching"; *κ*3 values are larger when branching is located at the extremities of the molecular graph or when no branching happens in the molecule, and they are smaller when branching is located near the center of the molecule [25]. Interestingly, the single ANN model that was selected for prediction of ab6A activity only used these two descriptors. Considering that the descriptor values of activators all lie inside a very small range in which no non-activators are present (data not shown), and the fact that the selected ANN model has two hidden neurons, the network simply "cut out" the value range in which the activators of ab6A lie, a typical effect of overtraining. This may be a possible explanation for the rather poor predictive performance of the ab6A model. The ab6A ORN shows a somewhat broader selectivity characteristic: activators are not as easy to discriminate from non-activators as for the other ORNs, and our method of assigning binary activity values may not have been appropriate in this case. Here it is important to note that ab6A is the only ORN in this study for which the receptor gene could not yet be identified [3].

#### General remarks

All these interpretations should be treated with care. It is not justified to interpret an individual descriptor as the sole discriminating feature. Rather, the KS-statistics demonstrate that many features are suitable for classification. Descriptor selection is the result of a statistical procedure, and depends on the composition of the data set. The results we present in this section should be read as an example of how to extract knowledge from such an analysis. Moreover, the ANN models combine the information obtained from the selected features to represent a more complex and nonlinear (except for Perceptron-type ANNs) relationship between molecular structure and activity than is suggested by the inherently linear descriptor ranking.

With these notes of caution, one might speculate that binding of odor molecules is achieved through different receptor-ligand interaction mechanisms at each OR. For example, our study suggests that ab2A is activated at least in part by the polarity of small ligands, whereas ab5B appears to require the flexibility of large ligands. While in the past the classification of chemical stimuli was based on selected functional groups or chemical class, the use of physicochemical descriptors provides a different view on the molecular features that govern ORN activation.

A systematic analysis of ORN selectivity was complicated by the limited amount of ORN response data. Only recently, more comprehensive data on *Drosophila *ORN responses became available [26]. Although the data was acquired using a different methodology (heterologous expression of OR genes in an "empty" ORN), it is possible that more data on these ORs will yield better results. This may be a fruitful task for a future study. It will be interesting to see if the abstract description of chemical entities as we used here can aid to reveal a logical structure in the selectivity of ORNs.

### Using ORN responses to predict ORN responses

If ORN responses really span some sort of chemical space, it should as well be possible to use the spike rates as a descriptor. To assess this hypothesis, we tried to predict activity of one ORN using responses of the remaining ORNs. We used the logarithm of the spike rates, because principle component analysis showed that this transformation results in a more uniform distribution with less outliers (data not shown). ANN training and model selection followed the same protocol as above, except that only 150 pairs of test and training data were formed and, no additional validation data was available to prune networks that showed poor generalization. Since only six descriptors were available to train the ANNs, we did not apply KS-statistics for data reduction.

The results are given as correlation coefficients in Table 2. ORNs ab3A, ab3B, ab5B, and ab6A show moderate correlation (MCC between 0.47 to 0.66) on the test set, but prediction completely failed for ab1D, ab2A (MCC = 0, respectively) and ab2B (MCC = -0.10). This indicates that this approach indeed works, at least for four out of seven receptors. The failure at the remaining three likely results from the fact that for these receptors there are too few actives in the test set, namely one for each ab1D and ab2A (salicylaldehyde and propyl acetate resp.), and three for ab2B (octanol, ethyl 3-hydroxybutanoate and 2-octanone).

**Table 2:** Matthew's Correlation Coefficient (MCC) for training and screening (test) using ORN responses as descriptor. The upper row refers to the performance on the training data, the lower row to performance on the test data

ORN		ab1D	ab2A	ab2B	ab3A	ab3B	ab5B	ab6A
MCC	training	0.55	0.0	0.76	1.00	0.77	0.80	0.72
	test	0.0	0.0	-0.10	0.47	0.66	0.54	0.54

## Conclusion

We have demonstrated that it is possible to predict *Drosophila *ORN responses from molecular structure. The approach performed well on the majority of receptors, considering that only few data was available for training. The features that were selected as being suitable for model training indicate that each ORN has different preferences regarding the physicochemical properties of its potential ligands. Finally, the ORN responses themselves can effectively be used as a descriptor to predict responses of other ORNs, providing evidence that ORNs indeed analyze chemical space in a way that can be exploited to predict receptor-ligand affinities.

## Experimental

We prepared a database containing the molecular structures of the odorants previously screened [18] and their activity (in spikes/s) on the neurons of the classes ab1D, ab2A, ab2B, ab3A, ab3B, ab5B and ab6A. We chose these ORNs because interpretation of the response spectrum was not complicated by high responses to the solvent, and at least four molecules were active for these ORNs. This yielded a minimum ratio of active to inactive molecules of roughly 1 to 10, and allowed splitting of the data into a training and a validation set of the same size, and at least two instances of active molecules in each set (cf. "Neural Network Training").

### Definition of activity ranges

We transformed the continuous range of activity levels [see Additional file 2] into all-or-none data by setting a lower and an upper threshold for each ORN. Molecules with activities below the lower threshold were considered inactive, while those with activities above the upper threshold were considered active. Odorants with an activity value between the two thresholds were excluded from the modeling process, because their activity cannot be determined with a high level of confidence. The dose-response curve of ORNs is a sigmoid, and small differences in odor delivery can in result in changes in the concentrations producing inconsistencies between the previously published results [18] and the recordings in this study, particularly for these "borderline" odors.

To determine the two thresholds we used the following procedure: Starting from activity histograms for each ORN, we estimated a lower threshold below which a molecule is considered inactive. Assuming that the activities of inactive compounds would be distributed around zero spikes/s (but without knowing the true distribution), we estimated the lower threshold to be where the first "gap" in the activity histogram distribution was located. Similarly, we estimated the upper threshold above which we considered molecules as being active. This procedure is illustrated in the supplement [see Additional file 7].

In one case, additional data [3] indicated that ethyl acetate, considered inactive at ab5B according to the threshold, may actually be a weak activator for the ab5B neuron. In consequence, we marked its activity as "unknown". The final data collection including the thresholds for activity assignment can be reviewed in the supplement [see Additional file 2].

### Descriptor calculation, selection and ranking

We calculated 203 molecular descriptors using MOE (Chemical Computing Group, Montreal) for each odorant molecule, including calculated physical properties, subdivided surface areas, atom and bond counts, Kier & Hall connectivity and shape indices, adjacency and distance matrices, pharmacophore features, partial charge indices, potential energies, surface area, volume and shape indices and conformation dependent charge indices.

Prior to descriptor calculation, we generated heuristic 3D conformations with CORINA (Molecular Networks, Erlangen, Germany). At this stage, we used one conformation per molecule. Subsequently, those conformations were refined by energy minimization using MOE's MMFF94x force field, a modified version of the MMFF94s force field [27]. Minimization was stopped at a gradient of 10^-5.^

#### Pruning unsuitable descriptors

Nine descriptors were discarded because they had zero variance across odor molecules. Some descriptors (e.g. the dipole moment) depend on the three-dimensional conformation of the molecule, which could lead to inconsistent modeling results for different conformations. Because we do not know which conformation of an odorant stimulates the ORN we sought to eliminate descriptors that vary strongly with 3D conformation.

To identify such strongly varying descriptors, we generated multiple conformers of all odorants using MOE's stochastic conformer generation functionality, using an energy cutoff of 5 kcal/mol. This resulted in a median nine conformers per molecule, with a maximum of 956 conformers for nonanal. For each descriptor the variance over all conformers of an odorant was calculated and scaled using the Fano Factor [28], FD=σDμD
 MathType@MTEF@5@5@+=feaafiart1ev1aaatCvAUfKttLearuWrP9MDH5MBPbIqV92AaeXatLxBI9gBaebbnrfifHhDYfgasaacH8akY=wiFfYdH8Gipec8Eeeu0xXdbba9frFj0=OqFfea0dXdd9vqai=hGuQ8kuc9pgc9s8qqaq=dirpe0xb9q8qiLsFr0=vr0=vr0dc8meaabaqaciaacaGaaeqabaqabeGadaaakeaacqWGgbGrdaWgaaWcbaGaemiraqeabeaakiabg2da9maalaaabaacciGae83Wdm3aaSbaaSqaaiabdseaebqabaaakeaacqWF8oqBdaWgaaWcbaGaemiraqeabeaaaaaaaa@361D@, with *σ*_*D *_the variance and *μ*_*D *_the mean of descriptor *D *over all conformations, without prior normalization. We calculated the mean *F*_*D *_of each descriptor over all molecules and ranked the descriptors accordingly. Data from preliminary experiments (not shown) suggested a set of descriptors that particularly affected prediction quality through conformational variation. We selected the one with the smallest Fano Factor, which was the "dipole" descriptor with *F*_*D *_= 0.03, and eliminated all 26 descriptors with a mean *F*_*D *_≥ 0.03.

#### Descriptor selection

Descriptors were ranked by their ability to separate active from inactive molecules. This ability was assessed using the Kolmogorov-Smirnov (KS) test [29]. The KS-test compares the distribution of two series of data samples *A *and *B *by comparing, for each potential value *x*, the fraction of values from *A *less than *x *with the fraction of *B *values less than *x*. The KS-value (*k*_*KS*_) is the maximum difference over all *x *values. For each ORN, the descriptor values of all active odorants provided *A*, while *B *was provided by the inactive odorants.

The KS-test was performed using MATLAB R14 (The MathWorks, Natick, MA). For the ranking we used the *p*-value of the KS-test, that is, the probability that *A *and *B *stem from the same distribution. High KS-values result in low *p*-values. Descriptors with low *p*-values were ranked highest. Note that the ranking is specific and unique for each ORN. This is because for each ORN, different molecules constitute the active and inactive population, and ind consequence the descriptor values for active and inactive molecules are differently distributed.

### Artificial neural network training

We trained multilayer feed-forward Artificial Neural Networks (ANNs) to predict the activity of odorant molecules. Such networks have been described in detail elsewhere [30,31]. Briefly, a network with *k *inputs, *j *neurons in the hidden layer, and *i *output neurons delivers the output Oiμ
 MathType@MTEF@5@5@+=feaafiart1ev1aaatCvAUfKttLearuWrP9MDH5MBPbIqV92AaeXatLxBI9gBaebbnrfifHhDYfgasaacH8akY=wiFfYdH8Gipec8Eeeu0xXdbba9frFj0=OqFfea0dXdd9vqai=hGuQ8kuc9pgc9s8qqaq=dirpe0xb9q8qiLsFr0=vr0=vr0dc8meaabaqaciaacaGaaeqabaqabeGadaaakeaacqWGpbWtdaqhaaWcbaGaemyAaKgabaacciGae8hVd0gaaaaa@3118@ in response to a pattern *μ *according to equation (1):

Oiμ=g(bi+∑jWij⋅g(bj+∑kwjk⋅ξkμ)),
 MathType@MTEF@5@5@+=feaafiart1ev1aaatCvAUfKttLearuWrP9MDH5MBPbIqV92AaeXatLxBI9gBaebbnrfifHhDYfgasaacH8akY=wiFfYdH8Gipec8Eeeu0xXdbba9frFj0=OqFfea0dXdd9vqai=hGuQ8kuc9pgc9s8qqaq=dirpe0xb9q8qiLsFr0=vr0=vr0dc8meaabaqaciaacaGaaeqabaqabeGadaaakeaacqWGpbWtdaqhaaWcbaGaemyAaKgabaacciGae8hVd0gaaOGaeyypa0Jaem4zaC2aaeWaaeaacqWGIbGydaWgaaWcbaGaemyAaKgabeaakiabgUcaRmaaqafabaGaem4vaC1aaSbaaSqaaiabdMgaPjabdQgaQbqabaGccqGHflY1cqWGNbWzaSqaaiabdQgaQbqab0GaeyyeIuoakmaabmaabaGaemOyai2aaSbaaSqaaiabdQgaQbqabaGccqGHRaWkdaaeqbqaaiabdEha3naaBaaaleaacqWGQbGAcqWGRbWAaeqaaOGaeyyXICTae8NVdG3aa0baaSqaaiabdUgaRbqaaiab=X7aTbaaaeaacqWGRbWAaeqaniabggHiLdaakiaawIcacaGLPaaaaiaawIcacaGLPaaacqGGSaalaaa@5972@

with *g*(*x*) the transfer function of the output and hidden layer neurons respectively (see eq. (2)), *b*_*i*_, *b*_*j *_the bias of the neurons, *W*_*ij *_the weight of the *j*th hidden neuron to the *i*th output neuron, *w*_*jk *_the weight of *k*th input neuron to the *j*th hidden neuron, and ξkμ
 MathType@MTEF@5@5@+=feaafiart1ev1aaatCvAUfKttLearuWrP9MDH5MBPbIqV92AaeXatLxBI9gBaebbnrfifHhDYfgasaacH8akY=wiFfYdH8Gipec8Eeeu0xXdbba9frFj0=OqFfea0dXdd9vqai=hGuQ8kuc9pgc9s8qqaq=dirpe0xb9q8qiLsFr0=vr0=vr0dc8meaabaqaciaacaGaaeqabaqabeGadaaakeaaiiGacqWF+oaEdaqhaaWcbaGaem4AaSgabaGae8hVd0gaaaaa@31B3@ the *k*th element of input pattern *μ*. We used a sigmoidal transfer function (equation (2)):

g(x)=11+e−x,
 MathType@MTEF@5@5@+=feaafiart1ev1aaatCvAUfKttLearuWrP9MDH5MBPbIqV92AaeXatLxBI9gBaebbnrfifHhDYfgasaacH8akY=wiFfYdH8Gipec8Eeeu0xXdbba9frFj0=OqFfea0dXdd9vqai=hGuQ8kuc9pgc9s8qqaq=dirpe0xb9q8qiLsFr0=vr0=vr0dc8meaabaqaciaacaGaaeqabaqabeGadaaakeaacqWGNbWzcqGGOaakcqWG4baEcqGGPaqkcqGH9aqpdaWcaaqaaiabigdaXaqaaiabigdaXiabgUcaRiabdwgaLnaaCaaaleqabaGaeyOeI0IaemiEaGhaaaaakiabcYcaSaaa@39D6@

where *x *is the net input of a neuron.

The MATLAB Neural Network Toolbox was used for ANN modeling, employing backpropagation training with a gradient descent algorithm as implemented in MATLAB's traingdx function [30].

Descriptor values were scaled to zero mean and unit standard deviation (autoscaling) prior to network training. We assigned a target value of 1 to active molecules and 0 to inactive molecules. By random permutation and subsequent splitting we formed 250 pairs of test and validation data, keeping the fraction of active to inactive molecules identical in both sets.

Network performance during training was assessed using the mean standard error (MSE, equation (3))

MSE(Oexpect,Opredict)=1S∑i=1S(Oexpect−Opredict)2,
 MathType@MTEF@5@5@+=feaafiart1ev1aaatCvAUfKttLearuWrP9MDH5MBPbIqV92AaeXatLxBI9gBaebbnrfifHhDYfgasaacH8akY=wiFfYdH8Gipec8Eeeu0xXdbba9frFj0=OqFfea0dXdd9vqai=hGuQ8kuc9pgc9s8qqaq=dirpe0xb9q8qiLsFr0=vr0=vr0dc8meaabaqaciaacaGaaeqabaqabeGadaaakeaacqqGnbqtcqqGtbWucqqGfbqrcqGGOaakcqWGpbWtdaWgaaWcbaGaeeyzauMaeeiEaGNaeeiCaaNaeeyzauMaee4yamMaeeiDaqhabeaakiabcYcaSiabd+eapnaaBaaaleaacqqGWbaCcqqGYbGCcqqGLbqzcqqGKbazcqqGPbqAcqqGJbWycqqG0baDaeqaaOGaeiykaKIaeyypa0ZaaSaaaeaacqaIXaqmaeaacqWGtbWuaaWaaabCaeaacqGGOaakcqWGpbWtdaWgaaWcbaGaeeyzauMaeeiEaGNaeeiCaaNaeeyzauMaee4yamMaeeiDaqhabeaakiabgkHiTiabd+eapnaaBaaaleaacqqGWbaCcqqGYbGCcqqGLbqzcqqGKbazcqqGPbqAcqqGJbWycqqG0baDaeqaaOGaeiykaKYaaWbaaSqabeaacqaIYaGmaaaabaGaemyAaKMaeyypa0JaeGymaedabaGaem4uamfaniabggHiLdGccqGGSaalaaa@6A2B@

where *O*_predict _was the output of the network and *O*_expect _was given by the target values.

The MSE on the training data served as fitness function during training. ANN training was stopped when the MSE on the validation data did not decrease for 5,000 training epochs.

### Model performance evaluation

Two factors greatly influence the outcome of ANN training: The ANN architecture (how many neurons to use in the hidden layer) and the number of inputs (molecular descriptors). More neurons in the hidden layer or a higher number of inputs to the ANN may allow for more complex description of the data, but the resulting model is also susceptible to overfitting, that is, modeling fine details without revealing the global data structure. Because these parameters are difficult to estimate in advance, we trained many networks with different combinations of parameters, varying the number of neurons in the hidden layer from one to four. In the special case of one hidden neuron, the ANN was reduced to a single neuron, which essentially is a Perceptron architecture [30]. To vary the number of descriptors, we cumulatively used the first 1, 2,...30 descriptors from the ranked list, meaning we used the first descriptor, then the first two and so on until we used all 30 highest-ranked descriptors.

In total, we trained 30,000 ANN models per ORN (4 architectures × 30 input dimensionalities × 250 repetitions with different data splitting). We proceeded with selection of models with high predictive quality in cross-validation. We used the Matthews Correlation Coefficient MCC [32] to assess prediction quality (eq. (4)):

MCC=P⋅N+O⋅U(N+U)⋅(N+O)⋅(P+U)⋅(P+O),
 MathType@MTEF@5@5@+=feaafiart1ev1aaatCvAUfKttLearuWrP9MDH5MBPbIqV92AaeXatLxBI9gBaebbnrfifHhDYfgasaacH8akY=wiFfYdH8Gipec8Eeeu0xXdbba9frFj0=OqFfea0dXdd9vqai=hGuQ8kuc9pgc9s8qqaq=dirpe0xb9q8qiLsFr0=vr0=vr0dc8meaabaqaciaacaGaaeqabaqabeGadaaakeaacqqGnbqtcqqGdbWqcqqGdbWqcqGH9aqpdaWcaaqaaiabdcfaqjabgwSixlabd6eaojabgUcaRiabd+eapjabgwSixlabdwfavbqaamaakaaabaGaeiikaGIaemOta4Kaey4kaSIaemyvauLaeiykaKIaeyyXICTaeiikaGIaemOta4Kaey4kaSIaem4ta8KaeiykaKIaeyyXICTaeiikaGIaemiuaaLaey4kaSIaemyvauLaeiykaKIaeyyXICTaeiikaGIaemiuaaLaey4kaSIaem4ta8KaeiykaKcaleqaaaaakiabcYcaSaaa@569E@

where *P *is the number of "true positives", that is, data instances that are active and have also been predicted active. *N *("true negatives") is the number of data instances that are inactive and have been predicted inactive. *O *denotes the number of "overpredicted" instances, predicted active in spite of being inactive, and *U *is the number of "underpredicted" instances, that is, active instances predicted inactive. During each training run, we recorded the MCC on the training data as well as on the validation data for this run.

#### Model selection

A well-trained, well-generalizing model will have a high MCC both on the training and validation data. Hence, we selected ANNs with a training MCC equal or greater than their validation MCC, differing by no more than 0.1. From all ANNs fulfilling these criteria, we selected those with the maximum training MCC. If the selection resulted in more than one ANN, we used all selected ANNs and combined their prediction values by averaging.

For some ORNs, additional odorant activity data was available from other sources [3,33], providing an additional selection constraint on the models (see Table 3). Models failing to correctly predict the additional activity data were discarded. Of the additional compounds, Ethyl-3-hydroxybutyrate, a strong activator for ab3A according to [3], was not tested in [18], making it suitable as an additional validation point. Ethyl acetate was weakly active in [3] at the ab5B ORN but inactive in the original data. Assuming that it truly is an activator of ab5B, we excluded it from network training and used it to validate the ANN predictions. The remaining compounds in Table 3 were originally excluded from training because their activity fell in between the upper and lower activity threshold and thus could not be derived with certainty. Since the additional sources suggest they are active, we used them as validation compounds for model selection.

**Table 3:** Additional validation compounds. Additional odorant activity data we used for model selection. Sources: a: [33], b: [3]

ORN	Odorant Name	Source	Remarks
ab1D	furfural	a	-
ab2B	cyclohexanol	a	-
	(R)-ethyl-3-hydroxybutyrate	b	unknown stereoisomer, not tested in previous study [18]
ab3A	butyl acetate	b	-
	ethyl acetate	b	unsure activity in [18]
	1-hexanol	b	unsure activity in [18]
ab3B	pentyl Acetate	b	unsure activity in [18]
	E2-hexenal	b	unsure activity in [18]
ab5B	ethyl acetate	b	considered inactive in [18]

### Electrophysiology

We used the models to predict activity for a new set of odorants and tested the predictions in a new set of measurements from *Drosophila *ORNs. Electrical activity was recorded extracellularly by inserting glass electrodes into individual sensilla on the antenna of *Drosophila melanogaster *males as previously described [18,34]. Each sensillum houses several ORNs, either 4 (ab1 sensilla) or 2 (ab2, 3, 4, 5 and 6 sensilla). Neuronal excitation was measured as counts of spikes (action potentials) produced during a 500 ms stimulation period. Spike rates for each odorant were averaged from at least 9 (ab1 and ab2 sensilla), 7 (ab3 sensillum) or 3 individuals (ab5 and ab6 sensilla). It has previously been shown that spikes produced by the neurons in each of these sensilla can be reliably separated based on amplitude and shape differences [18,33,35]. The models were based on data generated with Tungsten electrodes but tested using saline filled glass electrodes. Both are standard methods that have been shown to produce similar results [34]. The fly was permanently bathed in a 194 cm/s air-stream. Most odorants were dissolved at 1% v/v in paraffin oil and air from a 5 ml syringe, containing 10 *μ*l on a small piece of filter paper, was injected with a ca. 9-fold dilution factor [18]. Three odorants were tested at a 100 times lower concentration [see Additional file 4] because they were extremely potent activators for some ORNs.

### Odorants

Odorants were obtained from Sigma, Aldrich or Fluka, of purity > 99% or highest available, except for octanal (98%), salicylaldehyde (98%), ethyl 3-hydroxybutanoate (98%) and 2-octanone (98%). Except for (S)-(+)-carvone, all chiral odorants were applied as racemic mixtures.

## Supplementary Material

Additional File 1 **trainingCompounds**. Odorant molecules for which ORN responses were obtained in [18]. Compound names are given in [Additional file 2]. Click here for file

Additional File 2 **trainingResponses**. Activity values (in spikes/s) and per-ORN thresholds. Spike rates of "active" odorants are set in bold in the respective column. Compounds in brackets have uncertain activity (i.e. spike rates between the upper and lower threshold). Click here for file

Additional File 3 **testCompounds**. These odorants were screened to check prediction quality. Compound names are given in [Additional file 4]. Click here for file

Additional File 4 **testResponses**. Measured response in test (t, in spikes/s) and predicted activation (p, 1 = predicted active, 0 = predicted inactive). The upper ten compounds were also part of the training set. Underlined ORN responses are considered active, those in plain font inactive according to the thresholds in ORN response [see Additional file 2]. Comparisons between predictions and measurements are marked up according to the following: --means true negative, ++ true positive, o false positive (overpredicted) and u false negative (underpredicted). Responses to ethyl-3-Hydroxybutyrate at ab2B and butyl acetate at ab3A have not been taken into account for the MCC calculation. Click here for file

Additional File 5 **pValues**. The ranked list of descriptors for each ORN and the associated *p*-values. Click here for file

Additional File 6 **descriptorMeaning**. The descriptors that we used in this study and how they are derived from chemical structure. Click here for file

Additional File 7 **threshold**. Binarizing activity using thresholds, here with respect to the ab3A ORN class. On the left, histogram representation of the activities, using 40 bins on the activity range. On the right, odorants arranged by activity (in spikes/s), with the highest activities most right. The two lines indicate the thresholds we determined. Odorants with activities below the lower threshold are considered "inactive" (blue circles), those above the upper threshold are considered "active" (red circles). The remaining odorants are excluded from the analysis (black circles). Click here for file
