# Supplementary material for: Predicting olfactory receptor neuron responses from odorant structure
Source: Chem Cent J. 2007 May 4;1:11. doi: 10.1186/1752-153X-1-11 (PMC1994056; doi:10.1186/1752-153X-1-11)
Supplement: Additional File 1 — trainingCompounds. Odorant molecules for which ORN responses were obtained in [18]. Compound names are given in [Additional file 2]. [file 1752-153X-1-11-S1.pdf]

Schmuker, de Bruyne, Hähnel and Schneider

Predicting olfactory receptor neuron responses from odorant structure — additional file 1

Odorant molecules for which ORN responses were obtained in (1). Compound names are given in additional file 2: trainingResponses.xls.

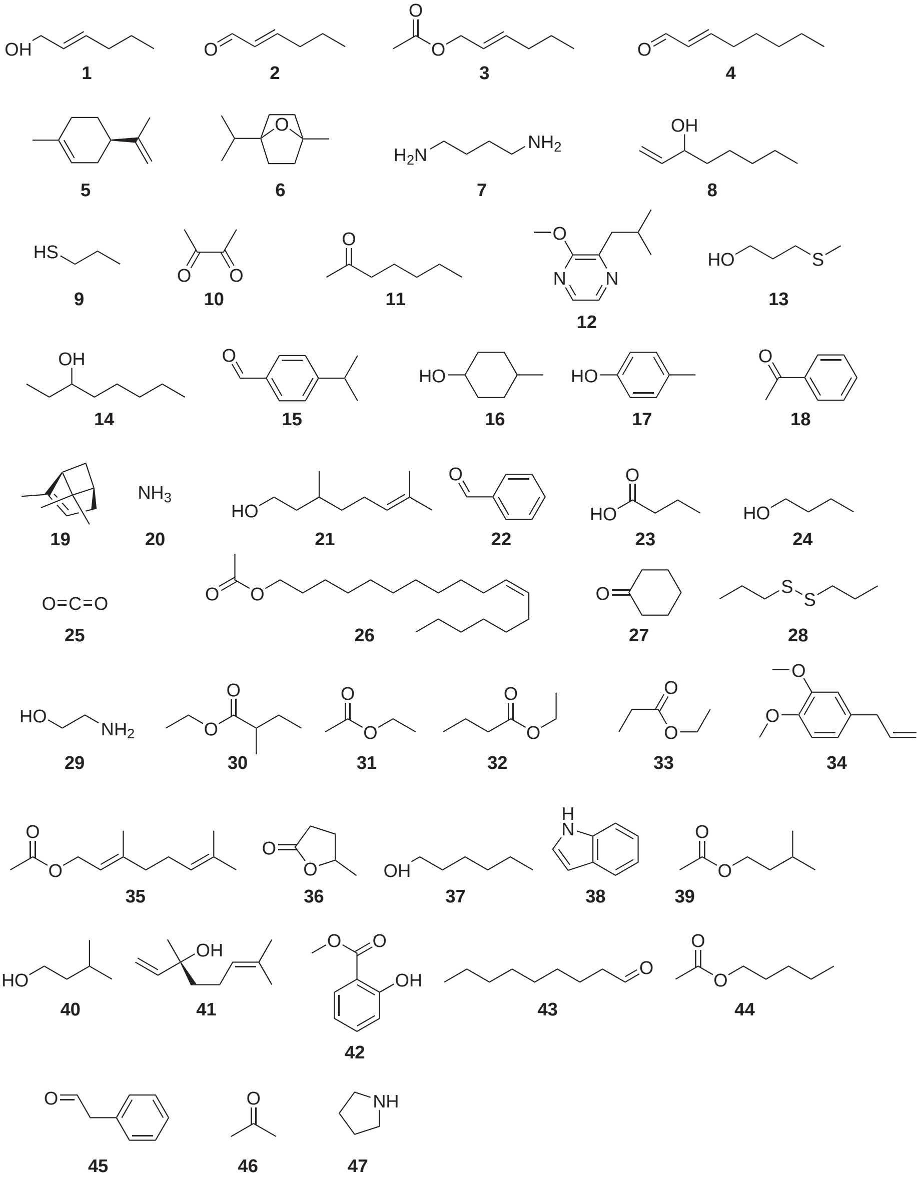

(1) de Bruyne M, Foster K, Carlson JR: **Odor coding in the *Drosophila* antenna.** *Neuron* 2001, **30**(2):537–552.
