# Supplementary material for: Predicting olfactory receptor neuron responses from odorant structure
Source: Chem Cent J. 2007 May 4;1:11. doi: 10.1186/1752-153X-1-11 (PMC1994056; doi:10.1186/1752-153X-1-11)
Supplement: Additional File 2 — trainingResponses. Activity values (in spikes/s) and per-ORN thresholds. Spike rates of "active" odorants are set in bold in the respective column. Compounds in brackets have uncertain activity (i.e. spike rates between the upper and lower threshold). [file 1752-153X-1-11-S2.pdf]

Schmuker, de Bruyne, Hähnel and Schneider

Predicting olfactory receptor neuron responses from odorant structure – additional file 2

index	substance name	ab1D	ab2A	ab2B	ab3A	ab3B	ab5B	ab6A
1	(E)2-hexen-1-ol	-3	**-3**	**56**	**2**	**16**	**0**	**123**
2	(E)2-hexenal	1	**-1**	**2**	**3**	(24)	**2**	**85**
3	(E)2-hexenyl acetate	-3	**8**	0	**114**	-11	**22**	**78**
4	(E)2-octenal	1	**-3**	**3**	(20)	8	**3**	**71**
5	(R)-(+)-limonene	-2	-7	**1**	-4	-6	-8	**-8**
6	1,4-cineole	-4	**-2**	**1**	**-1**	3	**0**	**-6**
7	1,4-diaminobutane	-1	-11	-1	**-1**	**19**	(13)	**3**
8	1-octen-3-ol	0	**-3**	(14)	**49**	**39**	(13)	**175**
9	1-propanethiol	3	-11	**2**	**5**	(22)	**7**	**-1**
10	2,3-butanedione	2	**102**	**1**	(21)	**46**	-5	**42**
11	2-heptanone	0	**5**	**1**	**33**	**122**	**70**	**48**
12	2-isobutyl-3-methoxy-pyrazine	6	**-4**	-1	-6	2	**-4**	**-3**
13	3-(methylthio)-1-propanol	9	-7	**4**	**2**	10	**152**	**14**
14	3-octanol	2	**-2**	**7**	**57**	**112**	**27**	**162**
15	4-isopropylbenzaldehyde	13	**-2**	**1**	-7	**16**	**-1**	**-14**
16	4-methylcyclohexanol	7	**0**	**3**	(13)	4	**-2**	**-15**
17	4-methylphenol	(22)	**-3**	0	**-1**	(31)	**1**	**-12**
18	Acetophenone	**157**	-8	**2**	-7	1	**1**	**-15**
19	alpha-pinene	-4	-8	0	-3	**13**	-5	**12**
20	Ammonia	5	-10	-1	**-1**	**17**	**5**	**-10**
21	beta-citronellol	-6	-7	**3**	-5	12	**4**	**53**
22	Benzaldehyde	49	-8	**1**	-5	3	**-1**	**-13**
23	Butanoic acid	1	**-2**	**1**	(20)	2	**-4**	**82**
24	Butanol	0	**13**	**2**	(11)	11	-5	**-16**
25	Carbon dioxide	0	**5**	**1**	**4**	**14**	**1**	**10**
26	*cis*-vaccenyl acetate	5	-6	**1**	(13)	-12	**-1**	**-16**
27	Cyclohexanone	-8	**-2**	-1	**3**	-1	**-2**	**-15**
28	Dipropyldisulphide	6	-10	-2	**2**	8	**5**	**4**
29	Ethanolamine	0	-9	**5**	**4**	9	**8**	**-6**
30	Ethyl 2-methylbutanoate	6	**14**	**23**	**141**	-7	**2**	**2**

31	Ethyl acetate	4	**156**	**1**	(14)	9	**8**	**9**
32	Ethyl butanoate	4	(23)	**73**	**145**	5	**3**	**-2**
33	Ethyl propionate	-1	**69**	(20)	**60**	-10	**8**	**-12**
34	Eugenol methyl ether	11	**-1**	**1**	-7	11	**-1**	**-15**
35	Geranyl acetate	2	**5**	0	**1**	(29)	-5	(26)
36	gamma-valerolactone	32	**-2**	**23**	**32**	-2	**-2**	**47**
37	Hexanol	3	**8**	**67**	(20)	**87**	**5**	**134**
38	Indole	4	**0**	**1**	**-1**	10	**-3**	**5**
39	Iso-amyl acetate	50	**7**	**9**	**104**	8	**45**	(24)
40	Iso-amyl alcohol	1	**1**	**4**	(14)	6	**1**	**-10**
41	Linalool	-2	-7	-4	**-1**	**14**	**-1**	(36)
42	Methyl salicylate	**187**	**-2**	**3**	**4**	3	**-3**	**5**
43	Nonanal	1	**-5**	-1	-4	6	**-4**	**-9**
44	Pentyl acetate	5	(23)	**2**	**111**	(25)	**198**	**69**
45	Phenylacetaldehyde	76	-6	0	-3	12	-5	-19
46	Propanone	-3	**88**	**1**	**1**	2	**1**	(35)
47	Pyrrolidine	2	**-5**	**6**	-4	(24)	**-1**	(28)
	lower threshold	20	20	10	10	20	10	20
	upper threshold	30	30	20	30	35	20	40
	number of actives	6	4	6	10	5	6	13
	number of inactives	40	41	40	28	36	39	29
