# Supplementary material for: Predicting olfactory receptor neuron responses from odorant structure
Source: Chem Cent J. 2007 May 4;1:11. doi: 10.1186/1752-153X-1-11 (PMC1994056; doi:10.1186/1752-153X-1-11)
Supplement: Additional File 3 — testCompounds. These odorants were screened to check prediction quality. Compound names are given in [Additional file 4]. [file 1752-153X-1-11-S3.pdf]

Schmuker, de Bruyne, Hähnel and Schneider

Predicting olfactory receptor neuron responses from odorant structure — additional file 3

These odorants were screened to check prediction quality. Compound names are given in additional file 4: testResponses.xls.

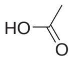
48

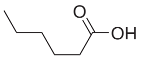
49

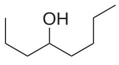
50

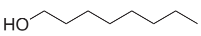
51

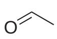
52

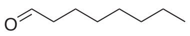
53

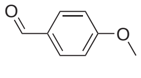
54

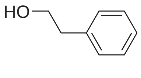
55

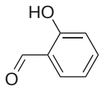
56

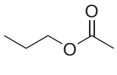
57

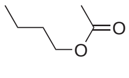
58

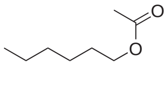
59

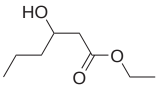
60

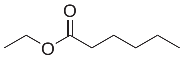
61

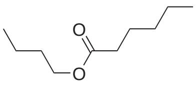
62

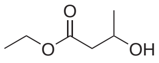
63

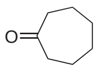
64

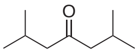
65

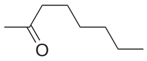
66

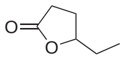
67

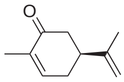
68
