# Supplementary material for: Predicting olfactory receptor neuron responses from odorant structure
Source: Chem Cent J. 2007 May 4;1:11. doi: 10.1186/1752-153X-1-11 (PMC1994056; doi:10.1186/1752-153X-1-11)
Supplement: Additional File 4 — testResponses. Measured response in test (t, in spikes/s) and predicted activation (p, 1 = predicted active, 0 = predicted inactive). The upper ten compounds were also part of the training set. Underlined ORN responses are considered active, those in plain font inactive according to the thresholds in ORN response [see Additional file 2]. Comparisons between predictions and measurements are marked up according to the following: --means true negative, ++ true positive, o false positive (overpredicted) and u false negative (underpredicted). Responses to ethyl-3-Hydroxybutyrate at ab2B and butyl acetate at ab3A have not been taken into account for the MCC calculation. [file 1752-153X-1-11-S4.pdf]

Schmuker, de Bruyne, Hähnel and Schneider

Predicting olfactory receptor neuron responses from odorant structure – additional file 4

Index	substance name	ab1D			ab2A			ab2B			ab3A			ab3B			ab5B			ab6A		
		t		p	t		p	t		p	t		p	t		p	t		p	t		p
23	butanoic acid	2	---	0	3	---	0	0	---	0	19	++	1	11	---	0	1	---	0	3	o	1
37	hexanol	5	---	0	6	---	0	53	++	1	(21)		1	95	++	1	6	---	0	168	++	1
14	3-octanol	0	---	0	1	---	0	2	---	0	40	++	1	124	++	1	13	o	1	222	++	1
22	benzaldehyde	32	++	1	3	---	0	2	---	0	8	---	0	14	---	0	0	---	0	11	---	0
17	4-methylphenol	5	o	1	1	---	0	-2	---	0	9	---	0	10	---	0	2	---	0	9	---	0
31	ethyl acetate	0	---	0	175	++	1	4	---	0	38	++	1	10	---	0	11	o	1	9	o	1
44	pentyl acetate	2	---	0	18	---	0	0	---	0	153	++	1	64	++	1	180	++	1	166	++	1
27	cyclohexanone	3	---	0	2	---	0	-1	---	0	33	u	0	(20)		0	2	---	0	12	---	0
10	2-heptanone	3	---	0	10	---	0	-2	---	0	58	++	1	207	++	1	62	++	1	126	++	1
35	geranyl acetate	2	---	0	(21)		0	-1	---	0	(19)		0	15	---	0	0	---	0	65	u	0
48	ethanoic acid	2	---	0	3	---	0	-2	---	0	9	o	1	16	---	0	1	---	0	12	o	1
49	hexanoic acid	0	---	0	3	---	0	1	o	1	33	++	1	11	o	1	3	o	1	4	o	1
50	4-octanol	1	---	0	0	---	0	4	---	0	53	++	1	8	o	1	(18)		0	82	++	1
51	octanol	7	---	0	6	---	0	58	u	0	(17)		1	18	o	1	6	---	0	59	u	0
52	acetaldehyde	2	---	0	1	o	1	0	---	0	9	---	0	10	---	0	1	---	0	0	---	0
53	octanal	3	---	0	2	---	0	2	---	0	9	---	0	(24)		1	4	---	0	(26)		0
54	4-methoxybenzaldehyde	(25)		1	4	---	0	1	---	0	6	---	0	18	---	0	1	---	0	(27)		0
55	2-phenylethanol	1	---	0	-3	---	0	4	---	0	(14)		0	12	---	0	2	---	0	11	---	0
56	salicylaldehyde	46	++	1	0	---	0	-1	---	0	7	---	0	16	---	0	1	---	0	17	---	0
57	propyl acetate	0	---	0	35	++	1	4	o	1	112	++	1	(29)		0	40	++	1	(27)		0
58	butyl acetate	4	o	1	17	---	0	-4	---	0	135		1	68	u	0	116	++	1	101	++	1
59	hexyl acetate	-1	---	0	9	---	0	-1	---	0	72	++	1	(33)		1	61	++	1	106	++	1
60	ethyl 3-hydroxyhexanoate*	1	---	0	5	---	0	9	o	1	57	++	1	17	---	0	47	u	0	(35)		0
61	ethyl hexanoate*	2	---	0	6	---	0	-1	---	0	217	++	1	3	o	1	1	---	0	3	---	0
62	butyl hexanoate	1	---	0	2	---	0	1	---	0	63	++	1	8	o	1	3	---	0	18	---	0
63	ethyl 3-hydroxybutyrate*	1	---	0	10	---	0	204		1	(14)		1	18	---	0	2	o	1	11	---	0
64	cycloheptanone	2	---	0	-2	---	0	-1	---	0	(27)		0	(20)		0	3	---	0	13	---	0
65	2,6-dimethyl-4-heptanone	2	---	0	1	---	0	40	u	0	(23)		1	14	---	0	4	---	0	12	---	1
66	2-octanone	1	---	0	3	---	0	3	---	0	37	++	1	153	++	1	34	++	1	160	++	1

67	gamma-hexalactone	4	---	0	6	---	0	-2	o	1	46	++	1	9	---	0	1	---	0	19	---	0
68	(S)-(+)-carvone	2	---	0	-5	---	0	-3	---	0	(11)		0	19	---	0	3	---	0	(29)		0

* These odorants were tested at a 100 times lower concentration
