# Supplementary material for: Predicting olfactory receptor neuron responses from odorant structure
Source: Chem Cent J. 2007 May 4;1:11. doi: 10.1186/1752-153X-1-11 (PMC1994056; doi:10.1186/1752-153X-1-11)
Supplement: Additional File 5 — pValues. The ranked list of descriptors for each ORN and the associated p-values. [file 1752-153X-1-11-S5.pdf]

Schmuker, de Bruyne, Hähnel and Schneider

Predicting olfactory receptor neuron responses from odorant structure – additional file 5

Rank	ab1D	*p*-value	ab2A	*p*-value	ab2B	*p*-value	ab3A	*p*-value	ab3B	*p*-value
1	std_dim3	0.0015	PEOE_VSA_FPNEG	0.0035	PEOE_VSA_FPPOS	0.0055	balabanJ	0.0001	SlogP_VSA8	0.0323
2	PEOE_VSA_FPNEG	0.0083	a_ICM		AM1_HOMO		MNDO_HF	0.0003	std_dim2	0.0429
3	a_ICM		PEOE_VSA_NEG		AM1_IP	0.0109	PEOE_VSA_FPPOS	0.00037	density	0.0460
4	dens	0.0124	apol		PEOE_RPC+	0.0124	FASA+	0.00044	PEOE_VSA_POS	0.0604
5	Q_VSA_FNEG		SMR_VSA5	0.0071	FASA+	0.0161	AM1_HOMO		dens	
6	Q_VSA_FPOS		chi1v_C		MNDO_HF	0.0233	PM3_HOMO		balabanJ	
7	FCASA-	0.0141	chi1_C		chi1_C		AM1_IP		PEOE_VSA+0	0.0645
8	Q_VSA_POS		PEOE_RPC+		PEOE_VSA_FHYD		PM3_IP	0.0007	PEOE_RPC-	
9	KierA3	0.0161	PEOE_VSA_HYD		PEOE_VSA_FPOL		MNDO_HOMO		PM3_HOMO	
10	Q_VSA_NEG		a_hyd		Q_VSA_FPPOS	0.0263	MNDO_IP	0.0010	PM3_IP	0.0836
11	FASA-	0.0206	SMR	0.0099	MNDO_HOMO		PEOE_VSA+5		Q_VSA_PPOS	
12	a_nH		chi0v_C		MNDO_IP		PEOE_VSA_POL	0.0014	Kier2	
13	b_1rotR		chi1v		PM3_HOMO		Q_RPC+		Kier3	0.0891
14	b_rotR	0.0233	PEOE_VSA_FHYD		PM3_IP	0.0334	RPC+	0.0016	AM1_HOMO	
15	density	0.0263	PEOE_VSA_FPOL		PEOE_VSA_PPOS		b_1rotR		AM1_IP	
16	SlogP_VSA7	0.0297	E_str		glob	0.0375	PEOE_VSA_PNEG		zagreb	0.1139
17	Kier3		mr		PM3_LUMO		PEOE_VSA_PPOS	0.0029	KierA2	0.1210
18	KierFlex		SlogP		FCASA+	0.0471	PEOE_VSA_POS		a_ICM	
19	SlogP_VSA4		logP(o/w)	0.0138	PEOE_VSA_FNEG		FCASA+	0.0039	KierA3	
20	glob	0.0334	a_nC		PEOE_VSA_FPOS	0.0526	SlogP_VSA2	0.0045	KierFlex	
21	vsa_acc	0.0375	chi0_C		Q_RPC+		SlogP_VSA1		SMR_VSA5	0.1617
22	a_aro		chi1		RPC+	0.0653	SMR_VSA0	0.0060	VDistMa	
23	b_ar	0.0420	PEOE_VSA-1		AM1_LUMO		PEOE_VSA+0	0.0091	b_1rotR	
24	b_rotN		rgyr		PEOE_PC+		a_nO		b_rotR	
25	E		vsa_hyd	0.0189	PEOE_PC-		PEOE_VSA_FHYD		Q_VSA_FPPOS	
26	bpol		weinerPath		PEOE_VSA_POL		PEOE_VSA_FPOL		MNDO_HOMO	
27	SMR_VSA3	0.0471	b_count		E_str		vsa_other	0.0118	MNDO_IP	0.2016
28	zagreb		Q_VSA_HYD		SMR_VSA6	0.0727	b_1rotN		VDistEq	
29	SMR_VSA0	0.0526	SlogP_VSA1		std_dim2	0.0894	b_rotR		b_1rotN	0.2127
30	PEOE_VSA+0	0.0587	vdw_vol	0.0256	Q_RPC-	0.0989	PEOE_VSA-5	0.0135	FCASA-	0.2242

ab5B	*p* -value	ab6A	*p* -value
KierA3	0.0010	Kier3	0.0003
KierA1		Kier2	0.0017
KierA2		balabanJ	0.0026
KierFlex	0.0064	VDistEq	0.0037
Kier2		SlogP_VSA8	0.0056
Kier3	0.0097	KierA3	0.0066
b_1rotR	0.0119	KierA2	0.0079
balabanJ		std_dim2	0.0098
rgyr		b_rotR	0.0158
std_dim1	0.0176	b_1rotR	0.0226
b_1rotN		KierFlex	0.0304
chi1v	0.0213	b_rotN	0.0351
Q_VSA_POS	0.0257	MNDO_HF	
b_rotN		FASA+	
MNDO_HF	0.0309	FCASA+	0.0368
VDistEq		petitjean	
PEOE_VSA+0	0.0370	petitjeanSC	
std_dim2	0.0440	MNDO_HOMO	
a_IC		MNDO_IP	0.0424
b_rotR		diameter	0.0557
bpol	0.0618	E	
glob	0.0728	PEOE_RPC+	0.0665
SMR_VSA7	0.0854	AM1_HOMO	
chi0v_C	0.0998	AM1_IP	
FASA+		Q_VSA_FPPOS	
PEOE_PC+		PM3_HOMO	
vsa_hyd	0.1161	PM3_IP	
PEOE_VSA_FPPOS		SlogP_VSA4	0.0757
PEOE_VSA_HYD		rgyr	0.0824
Kier1	0.1346	zagreb	0.0859
