# Supplementary material for: Predicting olfactory receptor neuron responses from odorant structure
Source: Chem Cent J. 2007 May 4;1:11. doi: 10.1186/1752-153X-1-11 (PMC1994056; doi:10.1186/1752-153X-1-11)
Supplement: Additional File 6 — descriptorMeaning. The descriptors that we used in this study and how they are derived from chemical structure. [file 1752-153X-1-11-S6.pdf]

Schmuker, de Bruyne, Hähnel and Schneider

Predicting olfactory receptor neuron responses from odorant structure — additional file 6

This table explains the meaning of the descriptors (adapted from the MOE user manual (Chemical Computing Group, Montreal, Canada)). Some descriptors occur in several variants, depending on the theory or algorithm underlying their calculation. For example, charge distribution for descriptors prefixed with Q was calculated using the MMFF94x force-field (1), while those prefixed with PEOE are based on calculations with the Partial Equalization of Orbital Electronegativities (PEOE) method proposed by Gasteiger and Marsili (2).

The following conventions are used in the table:
n: the number of atoms (not counting hydrogens);
m: the number of bonds (except bonds to hydrogen atoms);
a: the sum of $(r_i/r_c - 1)$ where r_i is the covalent radius of atom i, and r_c is the covalent radius of a carbon atom;
p_2: the number of paths of length 2 and p_3 the number of paths of length 3 in the molecular graph.

Molecular descriptors and their meaning.

Descriptor	Meaning
AM1_HOMO	Energy (eV) of the Highest Occupied Molecular Orbital calculated using the MOPAC AM1 Hamiltonian (3).
AM1_IP	Ionization potential (kcal/mol) calculated using the AM1 Hamiltonian (3).
AM1_LUMO	Energy (eV) of the Lowest Unoccupied Molecular Orbital calculated using the MOPAC AM1 Hamiltonian (3).
E	Value of the potential energy.
E_str	Bond stretch potential energy.
FASA+	Fractional ASA+ calculated as ASA+ / ASA.
FASA-	Fractional ASA- calculated as ASA- / ASA.
FCASA+	Fractional CASA+ calculated as CASA+ / ASA.
FCASA-	Fractional CASA- calculated as CASA- / ASA.
Kier2	Second kappa shape index: $(n-1)^2/m^2$ (4).
Kier3	Third kappa shape index: $(n-1)\cdot(n-3)^2/p_3^2$ for odd n, and $(n-3)\cdot(n-2)^2/p_3^2$ for even n (4).
KierA1	First alpha modified shape index: $s\cdot(s-1)^2/m^2$ where $s=n+a$ (4).
KierA2	Second alpha modified shape index: $s\cdot(s-1)^2/m^2$ where $s=n+a$ (4).
KierA3	Third alpha modified shape index: $(n-1)\cdot(n-3)^2/p_3^2$ for odd n, and $(n-3)\cdot(n-2)^2/p_3^2$ for even n where $s=n+a$ (4).
KierFlex	Kier molecular flexibility index: (KierA1)·(KierA2)/n (4)
MNDO_HF	Heat of formation (kcal/mol) calculated using the MNDO Hamiltonian (3).
MNDO_HOMO	Energy (eV) of the Highest Occupied Molecular Orbital calculated using the MNDO Hamiltonian (3).

Molecular descriptors and their meaning (*cont.*).

Descriptor	Meaning
MNDO_IP	Ionization potential (kcal/mol) calculated using the MNDO Hamiltonian (3).
PEOE_PC+	Total positive partial charge: the sum of the positive partial charges
{Q, PEOE}_RPC+	Relative positive partial charge: the largest positive q_i divided by the sum of the positive q_i
{Q, PEOE}_RPC-	Relative negative partial charge: the smallest negative q_i divided by the sum of the negative q_i
{Q, PEOE}_VSA+0	Sum of v_i where q_i is in the range [0.00,0.05)
{Q, PEOE}_VSA+5	Sum of per-atom van der Waals surface v_i where q_i is in the range [0.25,0.30)
{Q, PEOE}_VSA-1	Sum of v_i where q_i is in the range [-0.10,-0.05)
{Q, PEOE}_VSA_FHYD	Fractional hydrophobic van der Waals surface area
{Q, PEOE}_VSA_FNEG	Fractional negative van der Waals surface area
{Q, PEOE}_VSA_FPNEG	Fractional negative polar van der Waals surface area
{Q, PEOE}_VSA_FPOL	Fractional polar van der Waals surface area
{Q, PEOE}_VSA_FPOS	Fractional positive van der Waals surface area
{Q, PEOE}_VSA_FPPOS	Fractional positive polar van der Waals surface area
{Q, PEOE}_VSA_HYD	Total hydrophobic van der Waals surface area
{Q, PEOE}_VSA_NEG	Total negative van der Waals surface area
{Q, PEOE}_VSA_PNEG	Total negative polar van der Waals surface area
{Q, PEOE}_VSA_POL	Total polar van der Waals surface area
{Q, PEOE}_VSA_POS	Total positive van der Waals surface area
{Q, PEOE}_VSA_PPOS	Total positive polar van der Waals surface area
PM3_HOMO	Energy (eV) of the Highest Occupied Molecular Orbital calculated using the PM3 Hamiltonian (3).
PM3_IP	Ionization potential (kcal/mol) calculated using the PM3 Hamiltonian (3).
PM3_LUMO	Energy (eV) of the Lowest Unoccupied Molecular Orbital calculated using the PM3 Hamiltonian (3).
RPC+	Same as Q_RPC+
SMR	Molecular refractivity, calculated by an atomic contribution model (5)
SMR_VSA0	Sum of the approximate accessible van der Waals surface area v_i such that the contribution to Molar Refractivity for atom i (R_i) is in [0,0.11]
SMR_VSA5	Sum of v_i such that R_i is in (0.15,0.20]
SMR_VSA7	Sum of v_i such that $R_i > 0.56$
SlogP	Log of the octanol/water partition coefficient, calculated by an atomic contribution model (5)
SlogP_VSA1	Sum of v_i such that the contribution to logP(o/w) for atom i (L_i) is in (-0.4,-0.2]
SlogP_VSA2	Sum of v_i such that L_i is in (-0.2,0]
SlogP_VSA4	Sum of v_i such that L_i is in (0.1,0.15]
SlogP_VSA7	Sum of v_i such that L_i is in (0.25,0.30]
SlogP_VSA8	Sum of v_i such that L_i is in (0.30,0.40]
VDistEq	If m is the sum of the distance matrix entries then VdistEq is defined to be the sum of $\log_2 m - p_i \log_2 p_i / m$ where p_i is the number of distance matrix entries equal to i

Molecular descriptors and their meaning (*cont.*).

Descriptor	Meaning
VDistMa	If m is the sum of the distance matrix entries then VDistMa is defined to be the sum of $\log_2 m - D_{ij}\log_2 D_{ij}/m$ over all i and j.
a_ICM	Atom information content (mean). Let n_i be the number of occurrences of atomic number i in the molecule. Let $p_i = n_i/n$ where n is the sum of the n_i. The value of a_ICM is the negative of the sum over all i of $p_i \log p_i$.
a_IC	Atom information content (total). This is calculated to be a_ICM times n.
a_aro	Number of aromatic atoms
a_hyd	Number of hydrophobic atoms
a_nC	Number of carbon atoms
a_nH	Number of hydrogen atoms
a_nO	Number of oxygen atoms
apol	Sum of the atomic polarizabilities, with polarizabilities taken from (6)
b_1rotN	Number of rotatable single bonds (not including conjugated single bonds, such as petide and ester bonds)
b_1rotR	Fraction of rotatable single bonds
b_ar	Number of aromatic bonds
b_rotN	Number of rotatable bonds
b_rotR	Fraction of rotatable bonds
balabanJ	Balaban's connectivity topological index (7)
bpol	Sum of the absolute value of the difference between atomic polarizabilities of all bonded atoms in the molecule (including implicit hydrogens) with polarizabilities taken from (6)
chi0_C	Carbon connectivity index (order 0). This is calculated as the sum of $1/\sqrt{d_i}$, with d_i the number of bonded non-hydrogen atoms, over all carbon atoms i with $d_i > 0$
chi0v_C	Carbon valence connectivity index (order 0). This is calculated as the sum of $1/\sqrt{v_i}$ over all carbon atoms i with $v_i > 0$, with $v_i = (p_i - h_i)/(Z_i - p_i - 1)$ where p_i is the number of s and p valence electrons and Z_i the atomic number of atom i.
chi1	Atomic connectivity index (order 1) from (4) and (8). This is calculated as the sum of $1/\sqrt{d_i d_j}$ over all bonds between heavy atoms i and j where $i < j$
chi1_C	Carbon connectivity index (order 1). This is calculated as the sum of $1/\sqrt{d_i d_j}$ over all bonds between carbon atoms i and j where $i < j$
chi1v	Atomic valence connectivity index (order 1) from (4) and (8). This is calculated as the sum of $1/\sqrt{v_i v_j}$ over all bonds between heavy atoms i and j where $i < j$
chi1v_C	Carbon valence connectivity index (order 1). This is calculated as the sum of $1/\sqrt{v_i v_j}$ over all bonds between carbon atoms i and j where $i < j$
dens	Mass density: molecular weight divided by van der Waals volume (calculated using a grid approximation with spacing 0.75 Å)

Molecular descriptors and their meaning (*cont.*).

Descriptor	Meaning
density	Molecular mass density: Weight divided by the van der Waals volume (calculated using a connection table approximation)
diameter	Largest value in the distance matrix (9).
glob	Globularity, or inverse condition number (smallest eigenvalue divided by the largest eigenvalue) of the covariance matrix of atomic coordinates. A value of 1 indicates a perfect sphere while a value of 0 indicates a two- or one-dimensional object.
logP(o/w)	Log of the octanol/water partition coefficient, calculated from a linear atom type model implemented in MOE
mr	Molecular refractivity, calculated from an 11 descriptor linear model implemented in MOE
petitjean	Value of (diameter-radius) / diameter, with diameter the largest value in the distance matrix and radius defined as follows: If r_i is the largest matrix entry in row i of the distance matrix D, then radius is defined as the smallest of the r_i (9)
petitjeanSC	Petitjean graph Shape Coeffecient as defined in (9): (diameter-radius) / radius
rgyr	Radius of gyration
std_dim1	Standard dimension 1: the square root of the largest eigenvalue of the covariance matrix of the atomic coordinates. A standard dimension is equivalent to the standard deviation along a principal component axis
std_dim2	Standard dimension 2: the square root of the second largest eigenvalue of the covariance matrix of the atomic coordinates
std_dim3	Standard dimension 3: the square root of the third largest eigenvalue of the covariance matrix of the atomic coordinates
vsa_acc	Approximation to the sum of VDW surface areas of pure hydrogen bond acceptors (not counting acidic atoms and atoms that are both hydrogen bond donors and acceptors such as -OH)
Zagreb	Zagreb index: the sum of d_i^2 over all heavy atoms i, with d_i the number of non-hydrogen atoms to which atom i is bonded

References

[1] Halgren TA: **MMFF VI. MMFF94s option for energy minimization studies**. *Journal of Computational Chemistry* 1999, **20**:720–729.

[2] Gasteiger J, Marsili M: **Iterative Partial Equalization of Orbital Electronegativity - A Rapid Access to Atomic Charges**. *Tetrahedron* 1980, **36**:3219.

[3] Stewart JJP: *MOPAC Manual*. 7th 1993.

[4] Hall LH, Kier LB: *Reviews in Computational Chemistry*, Indianapolis: Purdue University at Indianapolis, *Volume 2* 1991 chap. The Molecular Connectivity Chi Indexes and Kappa Shape Indexes in Structure-Property Modeling, :367–422.

[5] Wildman S, Crippen G: **Prediction of Physicochemical Parameters by Atomic Contributions**. *Journal of Chemical Information and Modeling* 1999, **39**(5):868–873, [[http://dx.doi.org/10.1021/ci990307l]].

[6] CRC: *CRC Handbook of Chemistry and Physics*. CRC Press 1994.

[7] Balaban A: **Highly Discriminating Distance-Based Topological Index**. *Chemical Physics Letters* 1982, **89**:399–404.

[8] Hall LH, Kier LB: **The Nature of Structure-Activity Relationships and Their Relation to Molecular Connectivity**. *European Journal of Medicinal Chemistry* 1977, **12**:307–312.

[9] Petitjean M: **Applications of the Radius-Diameter Diagram to the Classification of Topological and Geometrical Shapes of Chemical Compounds**. *Journal of Chemical Information and Computer Science* 1992, **32**:331–337.
