# Supplementary material for: Predicting olfactory receptor neuron responses from odorant structure
Source: Chem Cent J. 2007 May 4;1:11. doi: 10.1186/1752-153X-1-11 (PMC1994056; doi:10.1186/1752-153X-1-11)

Schmuker, de Bruyne, Hähnel and Schneider

Predicting olfactory receptor neuron responses from odorant structure — additional file 3

Binarizing activity using thresholds, here with respect to the ab3A ORN class. On the left, histogram representation of the activities, using 40 bins on the activity range. On the right, odorants arranged by activity (in spikes/s), with the highest activities most right. The two lines indicate the thresholds we determined. Odorants with activities below the lower threshold are considered "inactive" (blue circles), those above the upper threshold are considered "active" (red circles). The remaining odorants are excluded from the analysis (black circles).

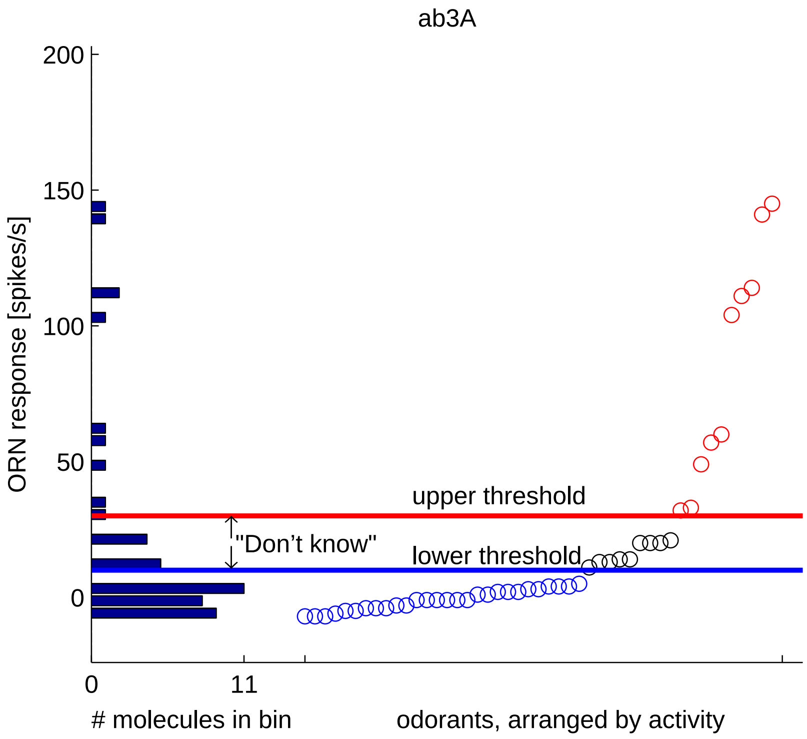

Supplement: Additional File 7 — threshold. Binarizing activity using thresholds, here with respect to the ab3A ORN class. On the left, histogram representation of the activities, using 40 bins on the activity range. On the right, odorants arranged by activity (in spikes/s), with the highest activities most right. The two lines indicate the thresholds we determined. Odorants with activities below the lower threshold are considered "inactive" (blue circles), those above the upper threshold are considered "active" (red circles). The remaining odorants are excluded from the analysis (black circles). [file 1752-153X-1-11-S7.pdf]
